# Strengths-based community action as a source of resilience for children affected by armed conflict

**DOI:** 10.1017/gmh.2015.23

**Published:** 2016-01-21

**Authors:** M. G. Wessells

**Affiliations:** Columbia University, Program on Forced Migration and Health, New York, USA

**Keywords:** Community action, interventions, resilience, strengths-based approach, war-affected children

## Abstract

Psychosocial and mental health supports for war-affected children frequently are limited by a deficits focus. Current research and practice indicate the value of a strengths-based approach that supports children's resilience and supports a positive environment for children and builds on existing strengths. This paper analyzes how community-based child protection mechanisms are a cornerstone of prevention efforts, and views community-based action as a particularly valuable source for strengths-based support for war-affected children. It shows how collective planning and action on behalf of vulnerable children create high levels of community ownership and effective, sustainable supports for children. It suggests that significant work lies ahead in strengthening the evidence base regarding the effectiveness of strengths-based approaches and in transforming practice away from expert-driven approaches toward community-driven action.

Globally, war exacts an enormous toll of suffering on children, who are defined under international law as people under 18 years of age. Mental health issues in war-affected children include depression, post-traumatic stress disorder (PTSD), and neurological problems, among others (Jones, 2008). Large numbers of children also suffer psychosocial distress stemming from problems such as family separation, displacement, loss of family and home, sexual abuse and violence, recruitment into armed forces or groups, trafficking, and HIV and AIDS, among others (Miller & Rasco, 2004; Boothby *et al*. 2006; Fernando & Ferrari, 2013). Lacking access to food, health care, education, or jobs, large numbers of conflict affected teenagers experience hopelessness about their future.

## Toward a strengths-based approach

Supports for war-affected children have frequently reflected a medical model that focuses primarily on deficits and entails treatment for problems such as PTSD or depression. To be sure, the provision of clinical supports for people who need specialized assistance is highly important, although clinical approaches are valuable mainly when they are part of a more comprehensive system of supports (IASC, 2007). However, it is a mistake to focus excessively on children's deficits. Large numbers of war-affected children exhibit remarkable resilience and actively cope with, adapt to, and navigate complex situations of adversity (Boothby *et al*. 2006; Wessells, 2006; Betancourt & Khan, 2008; Fernando & Ferrari, 2013; Tol *et al*. 2013*b*). Further, a deficits approach can lead one not to look for or to build upon the strengths that are present even in situations of adversity.

A stronger approach is strengths based and mobilizes existing resources and assets to support children's mental health and psychosocial well-being in war-affected contexts. Conceptually, this approach builds on the evidence that indicates children's resilience owes mostly to important protective and promotive factors in children's social ecologies (Boothby *et al*. 2006; Wessells, 2006; Ungar, 2012; Betancourt *et al*. 2013; Tol *et al*. 2013*b*). In the social ecologies of war-affected children, valuable strengths frequently include children's relations with significant other people–parents, extended family members, natural helpers, neighbors, peers, teachers, and religious leaders, among others.

A key to effective practice is to think not only about addressing deficits but to support and build upon these existing strengths in promoting children's well-being (IASC, 2007). Building on strengths can help to offset or mitigate the harms that are caused by accumulating risks (Rutter, 1979, 1985; Tol *et al*. 2013*b*). By drawing on existing strengths, it is possible to reach large populations of war-affected people before they develop mental health problems, thereby strengthening prevention (Tol *et al*. 2013*a*). Building on existing strengths also contributes to sustainability because the strengths are likely to persist even after the emergency mental health programs have ended (Wessells, 2015*a*). In contrast to dominant, deficits focused approaches, strengths-based approaches avoid stigmatizing war-affected children or implying that they are passive victims. Indeed, strengths-based approaches regard war-affected children as agents whose participation rights ought to be respected and who contribute via self-help to their own well-being (IASC, 2007; Wessells, 2015*a*).

## Community-level strengths and collective action

A community focus is useful for multiple reasons. Since most children and families live in groups or communities, community-level supports stand to reach large numbers of children. This is no small consideration in a war zone, where large numbers of children may have been affected and need support. The same applies in national settings such as the USA or the UK, particularly in areas where formal services are scarce or underutilized.

The multiple strengths that communities have tend to be sustainable and low-cost since they are typically not based on grants and outside specialists. These strengths involve, person-to-person support, typically by non-specialists. Quite often, the non-specialists consist of teachers, leaders, religious groups, women's groups, or youth groups, and cultural practices, among others (IASC, 2007). For example, in Southern Africa, where large numbers of children had been affected by political violence and orphaned by HIV and AIDS, faith-based groups organized supports for children that continued even without external support (Foster, 2004; Donahue & Mwewa, 2006). Similarly, in Sierra Leone, traditional Chiefs and elders frequently help to resolve inter-family conflicts over the responsibilities of a boy or man who has impregnated a girl (Wessells, 2011). Community strengths tend to persist, particularly when they are based on volunteer efforts and the commitment of natural helpers to support vulnerable children.

A community focus is useful also because children and families are not islands but need support from the communities in which they are embedded. Community-level actors are frequently positioned to help support vulnerable families or to intervene when problems such as severe child abuse arise. Working in different countries, I have seen respected religious leaders, for example, step in when severe family violence has erupted and harmed children.

Equally important, some of the greatest risks to children exist at the community level. Girls may be sexually harassed or raped as they walk to school. Boys or girls may be recruited into armed forces or into gangs near the areas where they live. Children may be living and working on the streets, outside of family care and often engaged in dangerous forms of labor. If children's mental health and well-being is to be promoted, community-level action to prevent such harms is surely needed.

Fortunately, communities are not passive in the face of such problems. Indeed, many communities exhibit considerable resilience and organize themselves for collective planning and action to address community problems. A case in point is community-based child protection mechanisms (CBCPMs), which are networks, groups, or mechanisms such as focal points that monitor risks to children, work to reduce or prevent those risks, provide informal support to children affected by the risks, and can, when appropriate, refer severely affected children to specialists (Wessells, 2009). CBCPMs can also report to authorities such as police cases in which, for example, children have been sexually violated and needs special protection and criminal prosecution of the perpetrator. In some cases, they may even report to authorities children who themselves have perpetrated criminal offenses.

International NGOs, which play a highly significant role in the field of child protection (Wessells, 2009; Child Protection Working Group, 2012), frequently facilitate the formation of CBCPMs in the form of Child Welfare Committees or Child Protection Committees. However, CBCPMs may also be community initiatives that embody local practices and values and arise without external facilitation. For example, a church in Malawi that is concerned about children who have become orphans owing to the HIV and AIDS pandemic might form an Orphans Support Group that provides housing, protection, and psychosocial support for orphans. Or, a Chief and his traditional court might address cases of relatively minor offences such as a teenager stealing food to help feed his family.

Existing evidence, which is still preliminary in nature, indicates that seven factors determine the effectiveness and sustainability of CBCPMs (Wessells, 2009). Two of the most important of these are: (1) building on local resources, and (2) local ownership. CBCPMs build on local resources or strengths to the extent that they link with and make use of strengths such as volunteer talent, the insights of natural helpers, peer support, local norms of helping one another, cultural practices or values (e.g. values such as *ubuntu*), and existing groups such as youth groups, women's groups, or religious groups. When CBCPMs build on local strengths, they serve as connectors that boost the effects of disparate resources and sharpen their focus to support children's well-being.

CBCPMS have local ownership to the extent that people see them as their own rather than as, for example, an NGO project, and regard them as their means of fulfilling their responsibility to support vulnerable children. In a community-owned CBCPM such as the Orphans Support Group mentioned above, local people's agency is at the center of the work, as local citizens have identified the problem, developed and implemented an approach for addressing it, and monitored its progress. Because it is ‘theirs,’ they take pride in its accomplishments and, when they encounter challenges, they use their ingenuity and local knowledge to solve them.

Community owned CBCPMs tend to be sustainable because they are not driven by outside money but by local people's values, motivation to support children, and sense of responsibility. An implication for the field of child protection, which is a preventive arm of children's mental health, is that greater emphasis should be placed on enabling high levels of community ownership. This is a tall challenge since the work of most NGOs is driven by technical experts rather than by communities, which at best are regarded as partners in a situation of unequal power (Miller & Rasco, 2004; Wessells, 2009, 2015*b*). In much humanitarian work, NGOs control the resources and power and guide the key decisions. As a result, NGO facilitated CBCPMs typically have only low-to-moderate levels of community ownership and tend to see a Child Welfare Committee as an ‘NGO project’ (Wessells, 2009, 2015*a*, *b*).

## Implications and challenges

An important implication for the field of child protection, and also for the wider field of mental health, is that greater emphasis needs to be place on local strengths and what communities themselves can do to support children's well-being. This requires a significant shift in both orientation and methodology in supporting mental health. Rather than viewing communities as loci for projects or mental health care activities (or even as ‘the problem’), we should view and engage with communities as actors who have strong agency and are in a position to mobilize local assets to support people's well-being. In regard to methodology, we should learn more deeply about the local assets that exist and avoid an excessive deficits focus. Further, we should devolve greater power to communities and support them as actors who can provide fundamental mental health and psychosocial supports. These implications also extend beyond the realm of war zones. In developing mental health systems even in highly developed societies, more attention should be given to building upon existing community strengths and enabling local groups and communities to support vulnerable people (Melton, 2009).

In order to make such a shift, however, significant challenges need to be addressed. For one thing, the evidence base regarding the effectiveness of community-driven, strengths-based interventions is relatively weak, and research using more robust designs and outcome measures is needed (Tol *et al*. 2011; Wessells, 2015*b*). Also needed is better evidence about how to enable strengths-based community action in settings that may not have well-defined ‘communities’ or where strengths are less abundant. Some urban settings, for example, have relatively low social cohesion and high levels of competition to meet basic needs, and they may have high population movement as well. Community action in such settings may be difficult and could be hijacked by a few powerful individuals (problems of power and exclusion arise also in stable, rural areas). In such settings, it may be essential to work in a manner that strengthens cohesion as well as builds on existing strengths.

An approach that places greater emphasis on community strengths and action also has important policy implications. In particular, child protection policies should prioritize, create space for, and support appropriate community action on behalf of vulnerable children. A promising development in Sierra Leone has been the Government's establishment in 2014 of a new Child and Family Welfare Policy that explicitly recognizes the importance of community action regarding children's protection and well-being and that District level Government should collaborate with and support appropriate community initiatives on behalf of children. At a grassroots level, communities are invited to take actions that support vulnerable children and collaborate with government actors. This bottom-up approach is a vital complement to the more typical top-down approaches to strengthening the national child protection system (Wessells, 2015*b*). It promises to help build community ownership of and support for formal child protection policies and services, align non-formal and formal aspects of the system, and use community strengths and agency to make the system more effective.

A significant challenge relates to the fact that many communities do not act to protect vulnerable children in ways that fit international child rights standards. In fact, they frequently have social norms that harm at risk children. For example, many communities in the war zones of sub-Saharan Africa and Asia have norms of early child marriage. In countries such as Somalia, parents marry their daughters at an age of 14 or 15 years in order to ‘protect’ them from being raped, which would spoil them for marriage and dishonor their families (Wessells *et al*. 2013). In such settings, it is vital to take a social norms change approach (Ahmed *et al*. 2009; Dagne, 2009) that works with and supports insider change agents who stir community change through slow processes of dialogue and in directions that support children's rights.

Perhaps the greatest challenge, though, will be in ourselves. To enable community-driven, strengths-based work, we need to listen more deeply to communities, relax our power and control, and focus less on our technical approaches than on supporting communities in promoting well-being. If we address these challenges, then we will move into a position to unleash the power of strengths-based community action approaches to strengthen mental health not only in war zones but throughout the world.
